# Impact of the European Union’s menthol cigarette ban on smoking cessation outcomes: longitudinal findings from the 2020–2021 ITC Netherlands Surveys

**DOI:** 10.1136/tc-2022-057428

**Published:** 2022-09-26

**Authors:** Christina N Kyriakos, Pete Driezen, Geoffrey Fong, Janet Chung-Hall, Andrew Hyland, Cloé Geboers, Anne C K Quah, Marc C Willemsen, Filippos T Filippidis

**Affiliations:** 1 Department of Primary Care and Public Health, School of Public Health, Imperial College London, London, UK; 2 Department of Psychology, University of Waterloo, Waterloo, Ontario, Canada; 3 School of Public Health Sciences, University of Waterloo, Waterloo, Ontario, Canada; 4 Ontario Institute for Cancer Research, Toronto, Ontario, Canada; 5 Health Behavior, Roswell Park Cancer Institute, Buffalo, New York, USA; 6 Department of Health Promotion (CAPHRI), Maastricht University, Maastricht, The Netherlands; 7 Netherlands Expertise Centre for Tobacco Control, Trimbos-institute, Utrecht, The Netherlands

**Keywords:** public policy, cessation, disparities

## Abstract

**Introduction:**

To reduce the appeal of tobacco, the European Union (EU) banned menthol as a characterising flavour in cigarettes in May 2020. This pre/post-study evaluated the impact of the menthol ban on smoking cessation outcomes among a representative cohort of Dutch smokers.

**Methods:**

Adult (18+ years) smokers were recruited at wave 1 (pre-ban) of the International Tobacco Control Netherlands Surveys (February–March 2020) and followed post-ban at wave 2 (September–November 2020) and wave 3 (June–July 2021) (N=1326 participated in all three waves). Weighted bivariate, logistic regression and generalised estimating equation model analyses were conducted.

**Results:**

Usual menthol use decreased from pre-ban (7.8%) to post-ban (4.0% at wave 2 and 4.4% at wave 3) (p<0.001). Pre-ban menthol smokers had greater odds of making a post-ban quit attempt than non-menthol smokers (66.9% vs 49.6%, adjusted OR (aOR)=1.89, 95% CI: 1.13 to 3.16). Compared with pre-ban non-menthol smokers, a higher proportion of menthol smokers quit by wave 2 (17.8% vs 10.2%, p=0.025) and by wave 3 (26.1% vs 14.1%, p=0.002), although this was not significant after adjusting for other factors. Female pre-ban menthol smokers had greater odds of quitting by wave 3 than female non-menthol smokers (aOR=2.23, 95% CI: 1.10 to 4.51). Most pre-ban menthol smokers (n=99) switched to non-menthol cigarettes (40.0%) or reported that they continued to smoke menthol cigarettes (33.0%) at wave 3.

**Conclusions:**

The EU menthol ban was effective in reducing menthol use and in increasing quit attempts and quitting among pre-ban menthol smokers. Impact could be maximised by closing gaps that allow post-ban menthol cigarette use.

**WHAT IS ALREADY KNOWN ON THIS TOPIC**

- The European Union (EU) banned menthol as a characterising flavour in cigarettes in May 2020. Pre/post-studies in Canada suggest that a menthol cigarette ban can increase quit attempts and quitting among menthol smokers compared with non-menthol smokers.

**WHAT THIS STUDY ADDS**

- This is the first known pre/post-study to examine the real-world impact of the EU menthol ban on cessation outcomes using a cohort of Dutch smokers followed from before to after the ban was implemented. Findings that the EU menthol ban resulted in 17.3% additional quit attempts and 12.0% additional quitting among menthol smokers as compared with non-menthol smokers in the Netherlands add to the evidence that banning menthol cigarettes can result in significant public health gains.

**HOW THIS STUDY MIGHT AFFECT RESEARCH, PRACTICE OR POLICY**

- Findings from this study can support policymakers, advocates and researchers in building the case for banning menthol cigarettes in other countries. It also highlights areas where the EU ban could be strengthened.

## Introduction

Menthol in cigarettes is well known to increase the appeal and attractiveness of smoking, which can facilitate initiation, sustain regular use and deter quitting.^1–3^ Through its unique sensory properties, including activating the ‘cooling’ receptors in the brain, menthol can mask the harshness of tobacco smoke on the throat and mouth, thereby making tobacco products more palatable, and inhalation easier.^4^ Menthol cigarettes have also contributed to widening health disparities, as they are used disproportionately by adolescents, women, racial/ethnic minority groups and other populations who have been selectively targeted by tobacco industry marketing.^1 5^

Partial guidelines for implementation of Articles 9 and 10 of the WHO Framework Convention on Tobacco Control (FCTC) recommend that Parties prohibit or restrict flavours in tobacco products.^6^ In May 2016, the European Tobacco Products Directive (TPD) banned characterising flavours in boxed cigarettes and roll-your-own (RYO) tobacco across the 27 European Union (EU) member states and the UK.^7^ However, there was a grace period until May 2020 for its application to products covering ≥3% of cigarette market sales volume, including menthol cigarettes.^7^ Other countries that have implemented menthol cigarette bans include Canada, Ethiopia, Moldova, Senegal and Turkey.^8^ Some countries have adopted, but not yet implemented menthol bans, such as Brazil, or are proposing legislation, such as the USA.^8^

Research suggests that banning menthol may reduce overall tobacco use, promote smoking cessation, prevent initiation, and substantially reduce tobacco-attributable deaths and life-years lost.^9–14^ Most of the research to date, however, has been disadvantaged by the lack of real-world menthol policies in place,^8^ consisting of simulation models,^13–16^ analyses of sales data,^17 18^ studies relying on expert elicitation to produce estimates of the impact of a hypothetical menthol ban,^19^ experimental studies,^20^ and survey studies assessing behavioural intentions of menthol smokers in response to a hypothetical or future ban.^21–24^ To date, population-level longitudinal studies of the actual behavioural impact of menthol bans have been conducted in Canada, where menthol cigarettes were banned between May 2015 and July 2017 among some provinces, progressing to a national ban in October 2017.^11 12 25–27^ Two pre/post-studies in Canada—the Ontario Menthol Ban Study^11^ and International Tobacco Control (ITC) Study^12^—found increased quit attempts,^11 12^ quitting^11 12^ and lower relapse^12^ after the menthol ban among daily menthol smokers compared with daily non-menthol smokers. In a pooled analysis of these two studies, menthol smokers overall were significantly more likely to have quit than non-menthol smokers (22.3% vs 15.0%; effect size: 7.3%, 95% CI: 2.1% to 12.5%, p=0.006).^28^

Within the European context, a few studies have examined menthol use and behaviours surrounding implementation of the TPD.^24 29–31^ Two studies using data from the 2016–2018 ITC surveys across eight European countries, including the Netherlands, examined menthol smokers’ anticipated responses to the menthol ban,^24^ as well as post-TPD cessation behaviours; however, this was conducted prior to the menthol ban going into effect.^29^ Another population-level survey study in England explored menthol cigarette use after the ban; however, data were cross-sectional and did not measure use prior to the ban.^30^ To the authors’ knowledge, no study to date has used cohort data to examine the pre/post-ban impact of the EU menthol cigarette ban. This study aims to evaluate the impact of the EU menthol cigarette ban on smoking cessation outcomes among a longitudinal sample of Dutch smokers.

## Methods

### Study design

Longitudinal data came from the ITC Policy Evaluation Netherlands Project with New Cohort 2020–2021 surveys. This is a three-wave prospective cohort study, part of the ITC Project cohort surveys, which have been conducted in 31 countries to examine the population-level impact of key policies of the WHO FCTC.^32^ At wave 1, adult smokers aged 18+ years were recruited (N=2067). The analytical sample was the longitudinal cohort of smokers recruited at wave 1 and retained at wave 2 and wave 3 (N=1326 respondents who participated in all three waves). The wave 1 survey was conducted from February to March 2020, before the May 2020 menthol cigarette ban was implemented (pre-ban). The wave 2 and wave 3 surveys were conducted from September to November 2020 and from June to July 2021, respectively, after the implementation of the menthol ban (post-ban).

Respondents were sampled from the TNS NIPObase, a database comprising more than 200 000 respondents randomly sampled from the Dutch population to participate in ongoing research by the survey firm, Kantar Public Netherlands. The sampling frame was designed to yield a representative random sample of smokers living in the Netherlands, within strata defined by age, sex and region. Sampling quotas were allocated proportionally to strata according to census data. Surveys were completed using computer-assisted web interviews, with an average survey length of 29.8 min.^33^ The response rate was 54.0%, with a cooperation rate of 93.9%.^33^ Further details on the methodology can be found elsewhere.^33^

### Measures

#### Pre-ban and post-ban menthol cigarette use

At recruitment (wave 1), respondents were adult smokers aged 18+ years who had smoked at least 100 cigarettes in their lifetime and smoked at least monthly. At post-ban (waves 2 and 3), smokers were defined as smoking at least monthly. Smokers were asked to report the flavour of their usual or current brand of cigarettes. Respondents who selected ‘menthol’ were classified as menthol smokers and those who reported ‘plain’ or ‘some other flavour’ were considered non-menthol smokers. Respondents who selected the option ‘don’t know’ at wave 1 (n=4) were set to missing. At post-ban, smokers who had reported a usual flavour at wave 1, but not at post-ban, were considered ‘smokers with flavour unknown’. Because this study was conducted by the ITC Project, measures and categorisations are identical or highly comparable with those used by Chung-Hall *et al* despite slight differences in wording of the measures used to assess menthol and non-menthol use in the ITC Canada Surveys (ie, ‘tobacco and menthol’ and ‘tobacco only’, respectively).^12^

#### Post-ban smoking cessation outcomes

Post-ban quitting at waves 2 and 3, respectively, was defined as self-reported quitting (no minimum length of abstinence specified in the survey) or smoking less than monthly, also comparable with Chung-Hall *et al*.^12^ Quit attempts were assessed using the question ‘How many serious quit attempts have you made since (last survey date)?’ Respondents were considered to have made a post-ban quit attempt if they reported having made one or more serious quit attempts at either wave 2 or wave 3, or if they were defined as having quit at either wave.

#### Covariates

Key sociodemographic covariates were region of residence (West, North, East, South), sex (male, female) and age group (18–24, 25–39, 40–54, 55+ years). Additionally, highest level of education was coded as low (primary education and lower pre-vocational secondary education), moderate (middle pre-vocational secondary education and secondary vocational education), high (senior general secondary education, pre-university education and higher professional education) and don’t know. Monthly household income was categorised as low (<€2000), moderate (€2000–3000), high (>€3000) and not stated.^34^ Smoking behaviours examined were smoking frequency (daily, non-daily); usual cigarette brand factory-made (FM) or RYO tobacco; nicotine dependence measured by the Heaviness of Smoking Index (HSI)^35^ (0–3; 4–6); ever tried to quit (yes, no) and plans to quit (no plans, plans within the next 6 months, plans in the future beyond 6 months).

### Statistical analysis

Bivariate and multivariable analyses were conducted in Stata/SE V.16.1 using weighted data with region as the stratum variable to account for the complex sampling design and for the oversampling of those aged 18–24 years old. Longitudinal inflation weights for the current cohort sample (those who participated in waves 1–3) were calibrated to represent the Dutch population of smokers at wave 1 by sex and age, education and region.^33^ Covariates for multivariable analyses were chosen because they were used to compute sampling weights,^33^ they were used in previous studies^32 36 37^ and for direct comparability with measures used by Chung-Hall *et al* 12 (except ethnicity, which was not measured in the ITC Netherlands Surveys and time-in-sample, which was not applicable here as the analytical sample participated in all three waves). In sensitivity analyses, covariates were included (or not) based on an iterative approach that also considered Akaike and Bayesian information criteria.

χ^2^ tests were conducted for bivariate comparisons between menthol and non-menthol smokers on wave 1 covariates (ie, sociodemographic variables and smoking behaviours). Comparisons between these groups were also made for post-ban outcomes using the overall sample, as well as stratified by sex. Bivariate results are presented as percentages with 95% CIs, per cent differences (% diff) and p values.

Binary generalised estimating equation (GEE) regression models (family: binomial; link: logit; correlation matrix: exchangeable) were used to assess changes in prevalence of usual menthol use between pre-ban (wave 1) and post-ban (waves 2 and 3), overall and by sex. GEE models adjusted for wave, region, sex (omitted in stratified analyses), age, household income, education (‘don’t know’ category set to missing, N=7), plans to quit within the next 6 months, ever made a quit attempt and HSI. Adjusted percentages and % diff are presented with 95% CIs and p values, computed using post-estimation margins commands in Stata.

Logistic regression models were used to examine the main effects between pre-ban menthol use and post-ban outcomes, overall and stratified by subpopulations (sex, age (18–39, 40+ years), household income, education, daily/non-daily smoking and HSI). To examine interaction effects, separate models (one model per interaction) were also fit to test the two-way interaction between flavour of usual brand and sociodemographic (sex, age, income, education) or smoking behaviour (smoking frequency, HSI) covariates. Sensitivity analyses were also conducted for other definitions of quitting and controlling for other covariates. Logistic regression analyses were adjusted for flavour of usual brand, sex, age (18–24, 25–39, 40–54, 55+ years), education, household income, plans to quit within the next 6 months, ever made a quit attempt and HSI at wave 1, and are presented as adjusted ORs (aORs) with 95% CIs and p values. In stratified analyses, the respective stratification variable was not controlled for in the models. Interaction models also adjusted for the interaction term.

## Results

### Sample characteristics by pre-ban menthol use

The sample consisted of 1326 respondents who were smokers at the time of recruitment and participated in all three waves of the ITC Netherlands 2020–2021 Surveys. At pre-ban (wave 1), 7.5% (n=99) of smokers reported that their usual brand was menthol. Compared with non-menthol smokers, a significantly higher proportion of menthol users were female, aged 25–39 years, had high education, only smoked FM cigarettes, smoked non-daily, had lower nicotine dependence and had plans to quit within the next 6 months (table 1). No differences between pre-ban menthol and non-menthol smokers were observed by region, household income and having ever made a quit attempt. Prevalence of menthol use overall and by sociodemographic characteristics and smoking behaviours across waves 1–3 are presented in online supplemental table 1. Characteristics of those who were lost to follow-up at waves 2 or 3 (N=741) compared with the cohort sample who participated in all three waves are shown in online supplemental table 2.

10.1136/tc-2022-057428.supp1 Supplementary data

**Table 1:** Wave 1 characteristics overall and by pre-ban menthol versus non-menthol use, among adult smokers who participated in waves 1–3 of the ITC Netherlands 2020–2021 Surveys,* weighted

Variable	Overall (N=1326)			Menthol smokers (n=99)			Non-menthol smokers (n=1223)			Menthol vs non-menthol
	n	%	95% CI	n	%	95% CI	n	%	95% CI	P value
Region										
West	547	46.5	46.1 to 46.9	52	57.0	47.4 to 66.1	494	45.7	44.8 to 46.6	0.060
North	154	11.5	11.3 to 11.8	11	11.8	6.7 to 19.9	142	11.5	10.9 to 12.0	
East	291	20.7	20.4 to 21.0	22	19.7	13.3 to 28.1	268	20.8	20.1 to 21.5	
South	334	21.2	19.4 to 23.1	14	11.5	6.9 to 18.6	319	22.0	21.4 to 22.6	
Sex										
Male	751	56.6	53.8 to 59.3	29	29.2	21.0 to 39.0	719	58.7	55.9 to 61.5	**<0.001**
Female	575	43.4	40.7 to 46.2	70	70.8	61.0 to 79.0	504	41.3	38.5 to 44.1	
Age group (years)										
18–24	166	14.2	12.3 to 16.0	13	17.2	10.3 to 27.2	153	14.0	12.0 to 16.2	**<0.001**
25–39	418	28.2	25.9 to 29.5	51	44.8	35.3 to 54.8	365	26.8	24.4 to 29.3	
40–54	363	27.0	24.6 to 29.5	22	24.3	16.6 to 34.2	340	27.2	24.8 to 29.8	
55+	379	30.6	28.1 to 33.2	13	13.6	8.0 to 22.1	365	32.0	29.4 to 34.7	
Household income										
Low	278	21.4	19.2 to 23.8	21	21.5	14.3 to 31.0	256	21.4	19.1 to 23.8	0.659
Moderate	258	19.4	17.3 to 21.7	16	15.6	9.6 to 24.1	242	19.8	17.6 to 22.2	
High	493	36.3	33.7 to 39.0	43	41.4	32.0 to 51.4	448	35.8	33.2 to 38.6	
Not stated	297	22.9	20.6 to 25.3	19	21.6	14.2 to 31.4	277	23.0	20.7 to 25.5	
Education										
Low	462	38.3	35.6 to 41.0	21	23.8	16.0 to 33.8	438	39.3	36.5 to 42.2	**<0.001**
Moderate	572	40.9	38.3 to 43.6	39	38.0	28.8 to 48.0	532	41.2	38.5 to 44.0	
High	285	20.3	18.3 to 22.6	38	37.3	28.2 to 47.4	247	19.0	16.9 to 21.3	
Not stated	7	0.46	0.22 to 1.0	1	0.9	0.1 to 6.2	6	0.4	0.2 to 1.0	
Smoking frequency										
Non-daily	202	15.0	13.2 to 17.1	25	25.3	17.5 to 35.0	176	14.1	12.3 to 16.2	**0.003**
Daily	1124	85.0	82.9 to 86.8	74	74.7	65.0 to 82.4	1047	85.9	83.8 to 87.7	
Usual brand										
FM	875	65.5	62.9 to 68.1	96	97.2	91.5 to 99.1	777	63.0	60.2 to 65.7	**<0.001**
RYO	446	34.5	31.9 to 37.1	3	2.8	0.9 to 8.5	441	37.0	34.3 to 39.8	
HSI										
Lower (0–3)	1037	78.8	76.5 to 81.0	84	86.5	78.3 to 92.0	948	78.2	75.7 to 80.5	0.051
Higher (4–6)	276	21.1	19.0 to 23.5	14	13.5	8.0 to 21.7	261	21.8	19.5 to 24.3	
HSI mean score	1313	2.2	2.1 to 2.3	98	1.5	1.2 to 1.8	1209	2.3	2.2 to 2.4	**<0.001**
Plans to quit										
No plans	129	12.4	10.5 to 14.6	6	6.3	2.8 to 13.6	123	13.0	11.0 to 15.3	**0.007**
Within 6 months	340	31.6	28.8 to 34.5	40	45.7	35.3 to 56.5	299	30.2	27.4 to 33.3	
In future >6 months	586	56.0	52.9 to 59.0	40	47.9	37.4 to 58.7	546	56.7	53.5 to 59.9	
Ever tried to quit										
No	314	24.0	21.8 to 26.5	20	21.5	14.2 to 31.2	293	24.2	21.8 to 26.8	0.565
Yes	1010	76.0	73.5 to 78.2	79	78.5	68.8 to 85.8	928	75.8	73.2 to 78.1	

Statistically significant values are indicated in bold.

*Wave 1 (pre-ban): February–March 2020; menthol ban: May 2020; wave 2 (post-ban): September–November 2020; wave 3 (post-ban): June–July 2021.

FM, factory-made cigarettes; HSI, Heaviness of Smoking Index; ITC, International Tobacco Control; RYO, roll-your-own tobacco.

### Pre/post-ban changes in adjusted prevalence of menthol cigarette use

The adjusted prevalence of usual menthol use among smokers significantly decreased from wave 1 (pre-ban) (7.8%, 95% CI: 6.3% to 9.4%) to wave 2 (post-ban) (4.0%, 2.8% to 5.2%) and wave 3 (post-ban) (4.4%, 3.1% to 5.6%). The adjusted GEE model showed that compared with wave 1, menthol use in the overall sample decreased by 3.8 percentage points at wave 2 (p<0.001) and by 3.5 percentage points at wave 3 (p<0.001). Among both women and men, respectively, menthol use significantly decreased from pre-ban to post-ban at wave 2 (% diff: −5.7 and −2.4) and at wave 3 (% diff: −4.4 and −2.9) (all p<0.001) (online supplemental table 3).

### Post-ban smoking cessation outcomes by pre-ban menthol use

#### Post-ban quit attempt

**Figure 1:**
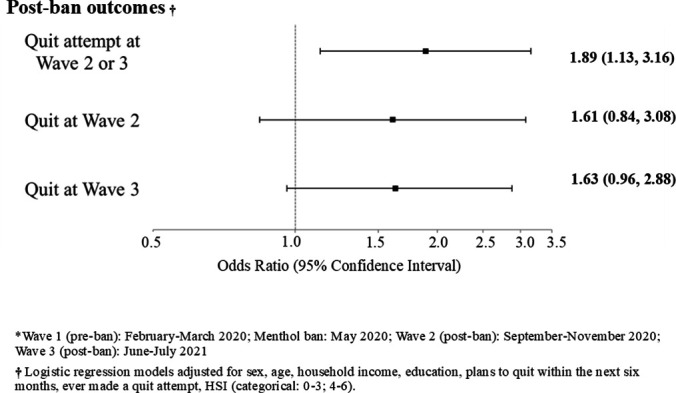
Post-menthol ban outcomes comparing pre-ban menthol smokers with non-menthol smokers (reference group), among respondents who participated in waves 1–3 of the 2020–2021 International Tobacco Control Netherlands Surveys*, weighted (N=1322).

Overall, 66.9% (56.2% to 76.1%) of menthol smokers made a post-ban quit attempt (wave 2 or 3) compared with 49.6% (46.5% to 52.8%) of non-menthol smokers (% diff: 17.3, p=0.002) (table 2 and online supplemental table 4). In adjusted logistic regression models, the odds of making a post-ban quit attempt were significantly higher among pre-ban menthol smokers than non-menthol smokers (aOR=1.89; 95% CI: 1.13 to 3.16, p=0.015) (figure 1). Menthol smokers were also significantly more likely to have made a post-ban quit attempt compared with non-menthol smokers among the following subgroups: men (aOR=3.00, 1.07 to 8.39), those aged 18–39 years (aOR=3.02, 1.31 to 6.97), those with high education (aOR=2.53, 1.01 to 6.33), daily smokers (aOR=2.15, 1.18 to 3.93) and those with higher nicotine dependence (aOR=6.97, 1.08 to 44.86). The only statistically significant interaction effect was between pre-ban menthol use and age (p=0.027) (online supplemental table 5).

**Table 2:** Post-ban smoking cessation outcomes by pre-ban usual menthol use, among respondents who participated in waves 1–3 of the 2020–2021 ITC Netherlands Surveys,* weighted (N=1322)†

Post-ban outcome	Pre-ban menthol smoker			Pre-ban non-menthol smoker (n=1223)			Comparison	
	(n=99)							
	n	%	95% CI	n	%	95% CI	% diff	P value
Quit attempt at wave 2 or 3	59	66.9	56.2 to 76.1	504	49.6	46.5 to 52.8	17.3	**0.002**
Quit at wave 2	17	17.8	11.2 to 27.0	127	10.2	8.6 to 12.1	7.6	**0.025**
Quit at wave 3	26	26.1	18.3 to 35.8	175	14.1	12.3 to 16.2	12.0	**0.002**

Statistically significant values are indicated in bold.

*Wave 1 (pre-ban): February–March 2020; menthol ban: May 2020; wave 2 (post-ban): September–November 2020; wave 3 (post-ban): June–July 2021.

†Excludes n=4 respondents who reported ‘don’t know’ as their usual brand flavour.

% diff, per cent difference; ITC, International Tobacco Control.

#### Post-ban quit

By wave 2, 17.8% (11.2% to 27.0%) of pre-ban menthol smokers had quit compared with 10.2% (8.6% to 12.1%) of non-menthol smokers (% diff: 7.6, p=0.025). By wave 3, 26.1% (18.3% to 35.8%) of pre-ban menthol smokers had quit compared with 14.1% (12.3% to 16.2%) of non-menthol smokers (% diff: 12.0, p=0.002) (table 2 and online supplemental table 4). Adjusted logistic regression analyses did not show statistically significant differences between pre-ban menthol and non-menthol smokers overall in having quit by wave 2 (aOR=1.61, 0.84 to 3.08) or by wave 3 (aOR=1.63, 0.96 to 2.88) (figure 1). However, female pre-ban menthol smokers had greater odds of having quit by wave 3 than female non-menthol smokers (aOR=2.23, 1.10 to 4.51). Moreover, among those who had moderate income, menthol smokers were significantly more likely to have quit by wave 2 than non-menthol smokers (aOR=4.50, 1.02 to 19.9). Interaction effects were not significant for any of the examined covariates (online supplemental table 5).

### Transitions in usual flavour and quitting from pre-ban to post-ban

**Figure 2:**
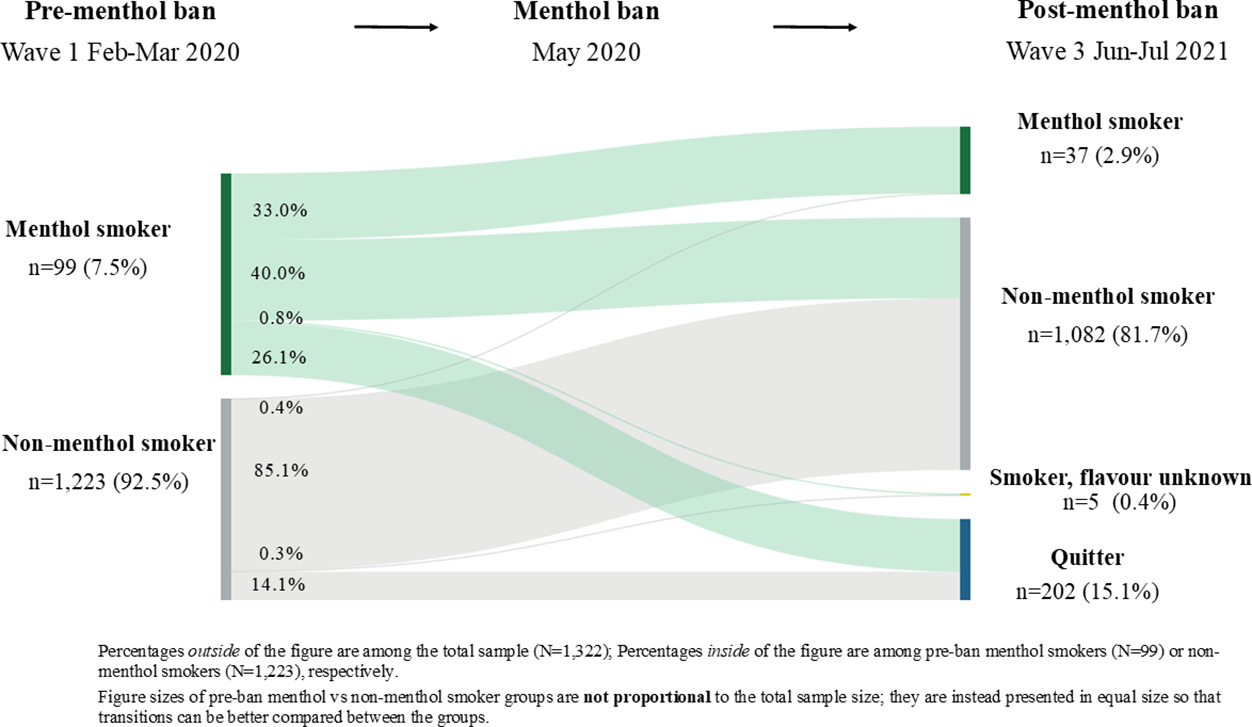
Transitions in smoking status from pre-menthol ban (wave 1) to post-menthol ban (wave 3) by pre-ban usual menthol use, among respondents who participated in waves 1–3 of the 2020–2021 International Tobacco Control Netherlands Surveys, weighted unadjusted (N=1322).

Figure 2 depicts transitions in smoking and quitting status from wave 1 (pre-ban) to wave 3 (post-ban) by pre-ban menthol use. Among pre-ban menthol smokers (n=99), 33.0% (24.3% to 43.1%) reported still smoking menthol cigarettes as their usual brand, 40.0% (30.7% to 50.2%) switched to non-menthol cigarettes as their usual brand, 0.8% (0.1% to 5.6%) remained smoking but with usual flavour unknown, and 26.1% had quit by wave 3. Among pre-ban non-menthol smokers (n=1223), 0.4% (0.2% to 1.1%) switched to menthol cigarettes, 85.1% (83.0% to 87.0%) continued to smoke non-menthol cigarettes, 0.3% (0.1% to 0.9%) continued to smoke but with no usual flavour reported, and 14.1% quit by wave 3.

## Discussion

This pre/post-study evaluated the EU menthol cigarette ban on smoking cessation outcomes among adult smokers in the Netherlands. Overall, we found that usual menthol use significantly decreased from pre-ban to post-ban. We also found a higher proportion of post-ban quit attempts and quitting among pre-ban menthol smokers compared with non-menthol smokers. After controlling for other factors, as compared with non-menthol smokers, menthol smokers had greater odds of making a post-ban quit attempt overall and among several subgroups, although the odds of post-ban quitting were only significant among women and among those with moderate income.

A strength of this study is its use of comparable measures, categorisations and adjusted covariates as the ITC Study by Chung-Hall *et al* that examined the impact of the Canadian menthol cigarette ban^12^; this comparability allows for more direct comparisons. Online supplemental table 6 presents comparisons of findings between the two studies. Overall, the findings from this study are largely consistent with results from Canada. Quit rates between menthol versus non-menthol smokers in our study (26.1% vs 14.1% at wave 3; % diff: 12.0) were higher than in Canada (21.5% vs 14.0%; % diff: 7.5).^12^ Larger effect sizes may be partly explained by the unique characteristics of menthol smokers in the Netherlands. Similar to what has been observed in other studies in Europe,^24 29 30^ the current sample of menthol smokers was more likely to be female, smoke FM cigarettes, be less nicotine dependent and smoke non-daily compared with non-menthol smokers. These differences were not observed in the ITC Canada Study.^12^ Moreover, according to 2016 Euromonitor Passport data, menthol cigarettes made up a larger share of the overall cigarette market in the Netherlands (4.7%) than in Canada (1.9%) prior to the bans.^38^ This is relatively low compared with countries with some of the highest menthol market shares (eg, 47.9% in Singapore and 28.6% in the USA).^38^ However, despite contextual differences between Canada and the Netherlands, the fact that both studies found comparable results of a positive impact of the menthol ban on cessation outcomes indicates that similar effects may be achieved in other countries.

We also found that female pre-ban menthol smokers were twice as likely to have quit than female non-menthol smokers; greater odds were also observed among those with moderate income. Moreover, the odds of making a post-ban quit attempt were higher among pre-ban menthol smokers compared with non-menthol smokers across many subgroups, notably among younger adults aged 18–39 years, daily smokers and those with higher nicotine dependence. While estimated interactions between covariates and pre-ban menthol use were not statistically significant (except for pre-ban menthol use by age in making a post-ban quit attempt), likely due to the small sample size, these findings suggest that the menthol ban may have been most successful among populations of highest risk, and therefore may help to advance health equity. This is of particular relevance to the USA, where a goal of the US Food and Drug Administration’s proposed tobacco product standard to prohibit menthol as a characterising flavour in cigarettes (announced on 28 April 2022) is to address tobacco-related health disparities.^39 40^ In the USA, menthol cigarettes are used disproportionately by non-Hispanic black smokers (85%).^41–43^

As also observed in Canada, we found that most pre-ban menthol smokers switched to non-menthol cigarettes, which is to be expected given the addictive nature of smoking.^12^ However, one-third of menthol smokers reported continuing to smoke menthol cigarettes after the ban, which is considerably higher than in Canada (19.5%).^12^ Consistent with this finding, a cross-sectional survey in England found that 15.7% of adult smokers reported smoking menthol cigarettes after the menthol ban (July 2020–July 2021).^30^

One possibility for reported post-ban menthol use is that smokers may be purchasing cigarettes on the market that are not overtly branded as having a ‘menthol’ flavour but may still be perceiving the product as mentholated. The EU ban still allows cigarettes to contain menthol (and other flavour) additives, but not at levels in which a characterising flavour(s) ‘other than that of tobacco’ is perceived as ‘clearly noticeable’ before or during smoking.^7^ This stands in contrast with the Canadian menthol ban that completely bans flavour additives.^8^ While tobacco manufacturers may have reduced the levels of menthol and other flavour additives and changed product names, it is plausible that even at lower levels or through use of other ‘non-flavour’ additives, some products may be imparting a characterising flavour or activating the ‘cooling’ receptors in the brain, thereby evoking sensory perceptions similar to that of menthol cigarettes. An investigative report through the *Organized Crime and Corruption Reporting Project* suggests that tobacco companies, such as Japan Tobacco International, have been exploiting challenges of determining and regulating ‘characterising flavours’.^44^ In the aftermath of the EU menthol ban, tobacco industry marketing materials pointed retailers and menthol smokers to ‘menthol replacement/alternative’ brands with ‘distinctive tobacco blends’.^44 45^ There is evidence that these products still contain high levels of menthol additives and have only been slightly rebranded from previous menthol cigarette brands.^44^ Moreover, as reported in Denmark, the tobacco industry continued to use brand descriptors and packaging after the ban to insinuate products as having ‘menthol-like qualities’, with several products accused of being in violation of the TPD.^46^

Another possible explanation for post-ban menthol use could be due to respondents reporting use of legal ‘flavour accessories’ (eg, separate capsules, RYO filters, flavour cards) to mentholate their cigarettes, or alternative menthol tobacco products (eg, cigarillos). While measurement of menthol cigarette use in this study was restricted to ‘usual brand of boxed cigarettes or RYO tobacco’, this definition may have been misinterpreted. Evidence suggests such flavour accessories and alternative products were introduced to the EU and UK markets, as well as in Canada, in an industry effort to undermine the menthol cigarette bans.^25 46–49^ Lastly, it is plausible that post-ban menthol use is due to illicit or cross-border purchasing, common industry arguments against menthol bans. However, this is not a likely explanation given that other policies in Europe have not resulted in increased availability of illicit cigarettes^50^ and there is no evidence of this in response to the Canadian menthol ban.^12 51^ Moreover, a survey study in England found declines in illicit or cross-border purchasing in the months following the menthol ban.^30^ The sizeable percentage of pre-ban menthol smokers still smoking menthol cigarettes at post-ban speaks to the unrealised additional gains from the menthol cigarette ban if this were addressed. Moreover, policy impact may be augmented by expanding the legislation to cover all menthol tobacco products and accessories and by adopting a complete additive ban. We plan to conduct future research examining post-ban menthol use.

This study has limitations that should be considered. First, even though the overall sample was large, the relatively small sample of pre-ban menthol smokers who were followed across all waves may have decreased statistical power to observe effects in some of the adjusted models. This also resulted in some subgroup analyses having wide CIs and likely attenuated statistical power in interaction models. Second, selection bias could have occurred due to differences between the analytical sample (those who participated in all three waves) compared with those who were lost to follow-up at waves 2 or 3, in which a higher proportion were female, aged 18–24 years, had high household income, had low education and were FM cigarette smokers. However, the two groups did not differ by the main predictor variable, flavour of usual brand or by other smoking behaviours (online supplemental table 2). Misclassification bias could have also occurred given that menthol use was defined as one’s usual cigarette brand, and therefore classified non-menthol users could have also been using menthol cigarettes simultaneously or occasionally. Moreover, quitting was defined based on self-report rather than biochemically verified abstinence, and included those who reported smoking less than monthly. However, sensitivity analyses using different definitions of quitting do not change main conclusions made in this study (online supplemental table 7).

Despite these limitations, a key strength of this study was the quasi-experimental design, in which we compared one group of smokers who was directly subjected to the menthol ban (menthol smokers) to another group who was not (non-menthol smokers) within the same country. An analysis of the difference between these two groups constituted a strong test of the impact of the menthol ban, increasing the internal validity of this policy evaluation study.^52^ Any alternative explanation for our finding that menthol smokers were more likely to quit than non-menthol smokers after the menthol ban must have an effect to increase quitting among menthol smokers compared with non-menthol smokers. We find it difficult to hypothesise such a causal factor that would lead to this difference in quit rates between menthol and non-menthol smokers other than the menthol ban. This is strengthened by the observation of higher quit rates among menthol versus non-menthol smokers only during the time period when the menthol ban was implemented.^29^ Prior to the EU menthol ban, from 2016 to 2018, ITC cohort studies across eight European countries, including the Netherlands, did not find significant differences in quit rates between menthol and non-menthol smokers (14.0% vs 12.0%).^29^

Findings support growing evidence of the substantial impact of a real-world menthol cigarette ban on encouraging smokers to quit.^11 12 26 27 53^ The high levels of increased quit attempts and quitting are of notable significance given that quitting behaviours are generally subpar in Europe.^36 54 55^ If the impact of 12.0% additional quitting found in this study were applied to the entire EU and UK, where 7.7% of adults were menthol smokers in 2017,^56^ increased quitting rates could mean more than a million additional quitters, which has considerable implications for averting smoking-related morbidity and mortality.

## Conclusions

The EU menthol ban was effective in reducing menthol use and in increasing quit attempts among pre-ban menthol smokers compared with non-menthol smokers. Higher levels of post-ban quitting were also observed; however, when accounting for other factors, this was only significant among women and those with moderate income. A substantial minority of smokers continued to report smoking menthol cigarettes at post-ban. In a context where the tobacco industry uses aggressive strategies to weaken menthol bans,^44 48 57^ addressing gaps in post-ban menthol use, such as through closing loopholes, strengthening compliance and increasing cessation support, is critical for maximising policy impact.

## Data Availability

Data are available upon reasonable request. In each country participating in the International Tobacco Control Policy Evaluation (ITC) Project, the data are jointly owned by the lead researcher(s) in that country and the ITC Project at the University of Waterloo. Data from the ITC Project are available to approved researchers 2 years after the date of issuance of cleaned data sets by the ITC Data Management Centre. Researchers interested in using ITC data are required to apply for approval by submitting an International Tobacco Control Data Repository (ITCDR) request application and subsequently to sign an ITCDR Data Usage Agreement. The criteria for data usage approval and the contents of the Data Usage Agreement are described online (http://www.itcproject.org).
