# Supplementary material for: Impact of the European Union’s menthol cigarette ban on smoking cessation outcomes: longitudinal findings from the 2020–2021 ITC Netherlands Surveys
Source: Tob Control. 2022 Sep 26;33(3):302–9. doi: 10.1136/tc-2022-057428 (PMC11041602; doi:10.1136/tc-2022-057428)
Supplement: Supplementary data [file tc-2022-057428supp001.pdf]

SUPPLEMENTARY TABLES

Supplementary Table 1. Prevalence of menthol use overall and by sociodemographic characteristics and smoking behaviours at Waves 1-3, among adult smokers who participated in Waves 1-3 of the 2020-2021 ITC Netherlands Surveys*, weighted, unadjusted (N=1,322)[1]

	Wave 1			Wave 2			Wave 3		
**Variable**	**n**	**%**	**95% CI**	**n**	**%**	**95% CI**	**n**	**%**	**95% CI**
**Overall menthol use**	**99**	**7.5**	**(6.2, 9.1)**	**45**	**3.6**	**(2.7, 4.9)**	**39**	**3.4**	**(2.5, 4.7)**
**Region**									
West	52	9.2	(7.1, 11.9)	23	4.5	(3.0, 6.7)	18	3.9	(2.5, 6.2)
North	11	7.7	(4.3, 13.6)	7	4.3	(2.0, 8.7)	6	4.3	(1.9, 9.5)
East	22	7.2	(4.7, 10.7)	7	2.5	(1.2, 5.3)	7	2.6	(1.2, 5.5)
South	14	4.1	(2.4, 6.9)	8	2.6	(1.3, 5.1)	8	2.7	(1.3, 5.4)
**Sex**									
Male	29	3.9	(2.7, 5.6)	14	1.9	(1.1, 3.3)	10	1.4	(0.8, 2.6)
Female	70	12.3	(9.8, 15.3)	31	5.8	(4.1, 8.2)	29	6.1	(4.2, 8.8)
**Age group (years)**									
18-24	13	9.1	(5.4, 15.1)	5	3.9	(1.6, 9.1)	6	5.4	(2.4, 11.6)
25-39	51	12.0	(9.2, 15.5)	24	6.0	(4.1, 8.9)	19	5.0	(3.2, 7.8)
40-54	22	6.8	(4.5, 10.1)	12	4.0	(2.3, 7.0)	11	4.0	(2.2, 7.1)
55+	13	3.3	(1.9, 5.7)	4	1.1	(0.4, 2.9)	3	0.7	(0.2, 2.2)
**Household income**									
Low	30	7.4	(5.2, 10.5)	12	3.0	(2.1, 5.8)	6	1.7	(0.8, 3.9)
Moderate	34	7.2	(5.1, 9.9)	15	3.5	(2.1, 5.8)	16	4.2	(2.6, 6.9)
High	16	9.8	(6.0, 15.5)	11	6.3	(3.5, 11.3)	11	6.9	(3.8, 12.3)
Not stated	19	7.1	(4.5, 10.9)	7	3.1	(1.4, 6.3)	6	2.6	(1.1, 5.8)
**Education**									
Low	21	4.7	(3.1, 7.1)	6	1.5	(0.6, 3.2)	9	2.4	(1.2, 4.6)
Moderate	39	7.0	(5.1, 9.5)	19	3.6	(2.3, 5.6)	16	3.1	(1.9, 5.0)
High	38	13.8	(10.1, 18.5)	19	7.4	(4.8, 11.5)	14	6.3	(3.7, 10.4)
Not stated	1	14.8	(2.0, 59.4)	1	14.8	(2.0, 59.4)	0	0.0	-
**Smoking frequency**									
Non-daily	25	12.7	(8.7, 18.3)	8	6.2	(3.1, 12.1)	9	7.4	(3.8, 13.9)
Daily	74	6.6	(5.3, 8.3)	32	3.0	(2.1, 4.2)	28	2.9	(2.0, 4.2)
**Usual brand FM or RYO**									
Factory-made (FM)	96	11.2	(9.2, 13.6)	43	5.4	(4.0, 7.3)	38	5.4	(3.9, 7.3)
Roll-your-own (RYO)	3	0.6	(0.2, 1.9)	2	0.5	(0.1, 2.0)	1	0.2	(0.0, 1.6)
**Heaviness of smoking index**									
Lower (0-3)	84	8.3	(6.7, 10.1)	32	3.6	(2.5, 5.1)	34	4.1	(2.9, 5.7)
Higher (4-6)	14	4.8	(2.8, 8.0)	7	2.5	(1.2, 5.3)	3	1.2	(0.4, 3.7)
Mean score	98	1.5	(1.2, 1.8)	39	1.6	(1.1, 2.1)	37	1.6	(1.1, 2.0)
**Plans to quit**									
No plans	6	4.2	(1.8, 9.1)	4	2.6	(1.0, 6.7)	5	4.2	(1.7, 9.9)
Within next 6 months	40	11.9	(8.8, 15.9)	18	5.4	(3.4, 8.5)	8	2.7	(1.3, 5.4)
In the future, >6 months	40	7.0	(5.1, 9.5)	18	3.4	(2.2, 5.5)	19	3.9	(2.5, 6.1)
**Ever tried to quit**									
No	20	6.8	(4.4, 10.3)	8	2.4	(1.2, 4.8)	10	3.2	(1.7, 6.0)
Yes	79	7.8	(6.3, 9.7)	37	4.1	(3.0, 5.7)	29	2.5	(2.5, 5.1)

*Wave 1 (pre-ban): February-March 2020; Menthol ban: May 2020; Wave 2 (post-ban): September-November 2020; Wave 3 (post-ban): June-July 2021; [1] Excludes n=4 respondents who reported "don't know" as their usual brand flavour.

Supplementary Table 2. Wave 1 characteristics of adult smokers who participated in Waves 1-3 (cohort sample) compared to those who participated in Wave 1 but were lost to follow-up at Waves 2 or 3 of the ITC Netherlands 2020-2021 Surveys*, weighted[1], unadjusted

	**Cohort sample (N=1,326)**			**Lost to follow-up (N=741)**			
**Variable**	**n**	**%**	**95% CI**	**n**	**%**	**95% CI**	**p-value**
**Region**							
West	547	46.7	(46.2, 47.1)	312	46.2	(45.7, 46.8)	0.887
North	154	11.4	(11.2, 11.7)	86	11.8	(11.4, 12.1)	
East	291	21.1	(20.7, 21.4)	151	20.0	(19.6, 20.5)	
South	334	20.8	(20.5, 21.1)	192	22.0	(21.6, 22.4)	
**Sex**							
Male	751	58.9	(56.2, 61.5)	378	52.2	(48.6, 55.9)	**0.004**
Female	575	41.1	(38.5, 43.8)	363	47.7	(44.1, 51.4)	
**Age group (years)**							
18-24	166	10.9	(9.4, 12.6)	165	20.3	(17.6, 23.2)	**<0.001**
25-39	418	26.8	(24.6, 29.2)	254	30.8	(27.6, 34.2)	
40-54	363	29.1	(26.7, 31.7)	154	23.1	(20.0, 26.4)	
55+	379	33.2	(30.6, 35.9)	168	25.8	(22.6, 29.3)	
**Household income**							
Low	278	30.0	(27.6, 32.6)	189	25.2	(22.2, 28.5)	**0.011**
Moderate	258	35.3	(32.7, 38.0)	135	18.6	(15.9, 21.6)	
High	493	12.2	(10.5, 14.1)	230	30.7	(27.4, 34.2)	
Not stated	297	22.5	(20.3, 24.9)	187	25.4	(22.4, 28.8)	
**Education**							
Low	462	37.3	(34.5, 39.9)	304	42.6	(39.0, 46.3)	**<0.001**
Moderate	572	40.0	(37.4, 42.7)	317	40.3	(36.8, 43.9)	
High	285	22.3	(20.1, 24.7)	108	15.4	(12.9, 18.4)	
Not stated	7	0.5	(0.2, 1.0)	12	1.6	(0.9, 2.7)	
**Smoking frequency**							
Non-daily	202	14.6	(12.8, 16.6)	108	13.8	(11.5, 16.5)	0.642
Daily	1124	85.4	(83.4, 87.2)	633	86.2	(83.5, 88.5)	
**Usual brand FM or RYO**							
FM	875	63.9	(61.2, 66.6)	537	71.2	(67.8, 74.4)	**0.001**
RYO	446	36.1	(33.4, 38.8)	202	28.8	(25.6, 32.2)	
**HSI**							
Lower (0-3)	1037	78.4	(76.0, 80.6)	583	78.7	(75.5, 81.2)	0.853
Higher (4-6)	276	21.6	(19.4, 24.0)	147	21.3	(18.3, 24.5)	
**HSI mean score** (0-6)	1313	2.2	(2.1, 2.3)	730	2.2	(2.1, 2.3)	0.347
**Plans to quit**							
No plans	129	13.0	(11.0, 15.3)	62	11.1	(8.7, 14.0)	0.314
Within the next 6 months	340	31.7	(28.9, 34.6)	213	34.9	(31.1, 38.9)	
In the future > 6 months	586	55.3	(52.2, 58.3)	319	54.0	(49.9, 58.1)	
**Ever tried to quit**							
No	314	22.9	(20.7, 25.3)	183	23.7	(20.8, 26.9)	0.699
Yes	1010	77.0	(74.7, 79.3)	556	76.3	(73.1, 78.2)	
**Flavour of usual brand**							
No	1223	92.8	(91.3, 94.1)	672	91.6	(89.4, 93.4)	0.335
Yes	99	7.2	(5.9, 8.7)	65	8.4	(6.6, 10.6)	

*Wave 1 (pre-ban): February-March 2020; Menthol ban: May 2020; Wave 2 (post-ban): September-November 2020; Wave 3 (post-ban): June-July 2021

[1] The sampling weight used in this analysis is designed to make these 2067 respondents representative of the adult (aged 18+) Dutch population of at least monthly cigarette smokers (and smoked at least 100 cigarettes in their lifetime) at the time of wave 1 data collection. The sampling weight used in all other analyses is also designed as such, however also adjusts for attrition between waves 2 and 3, hence, why proportions for the cohort sample varies slightly from Table 1.
FM=factory-made cigarettes; HSI=Heaviness of Smoking Index; RYO=roll-your-own tobacco

Supplementary Table 3. Changes in prevalence of usual menthol use before and after the menthol ban overall and by sex among adult smokers who participated in Waves 1-3 of the 2020-2021 ITC Netherlands Surveys*, weighted, adjusted

Wave	n	Adj % †	95% CI	% diff	95% CI	p-value
**Overall (N=1,322)**						
Wave 1 (pre-ban)	99	7.8	(6.3, 9.4)			
Wave 2 (post-ban)	45	4.0	(2.8, 5.2)	-3.8	(-5.1, -2.5)	**<0.001**
Wave 3 (post-ban)	39	4.4	(3.1, 5.6)	-3.5	(-4.9, -2.1)	**<0.001**
**Among females (N=575)**						
Wave 1 (pre-ban)	70	12.2	(9.4, 15.0)			
Wave 2 (post-ban)	31	6.5	(4.4, 8.7)	-5.7	(-8.1, -3.3)	**<0.001**
Wave 3 (post-ban)	29	7.8	(5.4, 10.3)	-4.4	(-6.8, -1.9)	**<0.001**
**Among males (N=751)**						
Wave 1 (pre-ban)	29	4.3	(2.7, 5.9)			
Wave 2 (post-ban)	14	1.9	(0.8, 2.9)	-2.4	(-3.8, -1.0)	**<0.001**
Wave 3 (post-ban)	10	1.4	(0.4, 2.3)	-2.9	(-4.5, -1.3)	**<0.001**

*Wave 1 (pre-ban): February-March 2020; Menthol ban: May 2020; Wave 2 (post-ban): September-November 2020; Wave 3 (post-ban): June-July 2021

† Generalised estimating equation (GEE) models (family: binomial, link: logit, correlation matrix: exchangeable) adjusted for sex, age, household income, education, plans to quit within the next six months, ever made a quit attempt, HSI (categorical: 0-3; 4-6), except for the respective stratification covariate; Post-estimation margins commands in Stata.

Adj= adjusted; % diff= percent difference

Supplementary Table 4. Post-ban quit attempt and quit by pre-ban usual menthol use overall and by sex, among respondents who participated in Waves 1-3 of the 2020-2021 ITC Netherlands Surveys*, weighted, unadjusted (N=1,322)

	**Post-ban quit attempt (Wave 2 or 3)**					**Post-ban quit (Wave 2)**					**Post-ban quit (Wave 3)**				
Pre-ban menthol use	**n**	**%**	**95% CI**	**% diff**	**p-value †**	**n**	**%**	**95% CI**	**% diff**	**p-value †**	**n**	**%**	**95% CI**	**% diff**	**p-value †**
**Overall (N=1,322)**															
Menthol	59	66.9	(56.2, 76.1)	17.3	**0.002**	17	17.8	(11.2, 27.0)	7.6	**0.025**	26	26.1	(18.3, 35.8)	12.0	**0.002**
Non-menthol	504	49.6	(46.5, 52.8)			127	10.2	(8.6, 12.1)			175	14.1	(12.3, 16.2)		
**Females (N=575)**															
Menthol	24	61.6	(48.8, 73.0)	12.8	0.065	12	17.4	(5.9, 10.7)	9.5	**0.012**	19	26.6	(17.4, 38.4)	14.2	**0.002**
Non-menthol	39	48.8	(44.0, 53.6)			42	7.9	(5.9, 10.7)			63	12.4	(9.7, 15.6)		
**Males (N=751)**															
Menthol	20	80.3	(60.3, 91.7)	30.1	**0.003**	5	18.5	(7.9, 37.6)	6.6	0.294	7	24.9	(12.3, 43.9)	9.5	0.175
Non-menthol	291	50.2	(46.1, 54.4)			85	11.9	(9.7, 14.5)			112	15.4	(12.9, 18.2)		

*Wave 1 (pre-ban): February-March 2020; Menthol ban: May 2020; Wave 2 (post-ban): September-November 2020; Wave 3 (post-ban): June-July 2021

† Chi-square test

% diff= percent difference

Supplementary Table 5. Main effects overall and by subpopulations and interaction effects between pre-ban usual menthol use and post-ban quit attempt and quit, among respondents who participated in Waves 1-3 of the 2020-2021 ITC Netherlands Surveys*, weighted, adjusted †

	Post-ban quit attempt (Wave 2 or 3)			Post-ban quit (Wave 2)			Post-ban quit (Wave 3)		
**Variable**	**aOR†**	**95% CI**	**p-value**	**aOR†**	**95% CI**	**p-value**	**aOR†**	**95% CI**	**p-value**
**Overall (N=1,322)**									
Menthol	1.89	(1.13, 3.16)	**0.015**	1.61	(0.84, 3.08)	0.149	1.63	(0.96, 2.88)	0.090
Non-menthol	1.00			1.00			1.00		
**Sex**									
**Females (N=575)**									
Menthol	1.74	(0.89, 3.41)	0.103	1.88	(0.84, 4.19)	0.123	2.23	(1.10, 4.51)	**0.026**
Non-menthol	1.00			1.00			1.00		
**Males (N=751)**									
Menthol	3.00	(1.07, 8.39)	**0.037**	0.87	(0.23, 3.32)	0.843	0.87	(0.26, 2.86)	0.818
Non-menthol	1.00			1.00			1.00		
**Interaction model: menthol x sex[1]**			0.336			0.233			0.188
**Age**									
**Aged 18-39 years (N=584)**									
Menthol	3.02	(1.31, 6.97)	**0.009**	1.34	(0.62, 2.86)	0.455	1.32	(0.67, 2.60)	0.418
Non-menthol	1.00			1.00			1.00		
**Aged 40+ years (N=742)**									
Menthol	1.08	(0.51, 2.28)	0.843	1.80	(0.51, 6.38)	0.360	2.00	(0.74, 5.42)	0.171
Non-menthol	1.00			1.00			1.00		
**Interaction model: menthol x age[1]**			**0.027**			0.817			0.810
**Household income**									
**Low income (N=278)**									
Menthol	2.66	(0.55, 12.86)	0.225	2.24	(0.44, 11.31)	0.330	1.33	(0.30, 5.82)	0.702
Non-menthol	1.00			1.00			1.00		
**Moderate income (N=258)**									
Menthol	4.06	(0.71, 23.18)	0.114	4.50	(1.02, 19.9)	**0.048**	2.79	(0.62, 12.52)	0.180
Non-menthol	1.00			1.00			1.00		
**High income (N=493)**									
Menthol	1.39	(0.70, 2.78)	0.344	0.98	(0.40, 2.42)	0.973	1.18	(0.55, 2.53)	0.669
Non-menthol	1.00			1.00			1.00		
**Income not reported (N=297)**									
Menthol	1.53	(0.38, 6.07)	0.547	0.63	(0.54, 7.47)	0.718	2.48	(0.51, 12.01)	0.260
Non-menthol	1.00			1.00			1.00		
**Interaction model: menthol x income[1]**			0.937			0.774			0.991
**Education**									
**Low education (N=462)**									
Menthol	2.25	(0.57, 8.92)	0.248	2.05	(0.41, 10.36)	0.385	1.56	(0.28, 8.58)	0.606
Non-menthol	1.00			1.00			1.00		
**Moderate education (N=572)**									

	Post-ban quit attempt (Wave 2 or 3)			Post-ban quit (Wave 2)			Post-ban quit (Wave 3)		
**Variable**	**aOR†**	**95%CI**	**p-value**	**aOR†**	**95%CI**	**p-value**	**aOR†**	**95%CI**	**p-value**
Menthol	1.85	(0.86, 3.96)	0.115	1.23	(0.40, 3.76)	0.719	1.76	(0.73, 4.26)	0.209
Non-menthol	1.00			1.00			1.00		
**High education (N=285)**									
Menthol	2.53	(1.01, 6.33)	**0.046**	1.30	(0.45, 3.81)	0.628	1.69	(0.72, 3.96)	0.225
Non-menthol	1.00			1.00			1.00		
**Interaction model: menthol x education[1]**			0.830			0.601			0.757
**Smoking frequency**									
**Daily smokers (N=1,124)**									
Menthol	2.15	(1.18, 3.93)	**0.012**	1.07	(0.41, 2.82)	0.890	1.44	(0.72, 2.88)	0.301
Non-menthol	1.00			1.00			1.00		
**Non-daily smokers (N=202)**									
Menthol	1.12	(0.29, 4.32)	0.872	2.18	(0.71, 6.70)	0.173	1.80	(0.56, 5.79)	0.326
Non-menthol	1.00			1.00			1.00		
**Interaction model: menthol x frequency[1]**			0.077			0.305			0.961
**Heaviness of smoking index (HSI)**									
**Lower HSI (0-3) (N=1,037)**									
Menthol	1.65	(0.95, 2.84)	0.073	1.79	(0.91, 3.54)	0.091	1.53	(0.83, 2.82)	0.177
Non-menthol	1.00			1.00			1.00		
**Higher HSI (4-6) (N=276)**									
Menthol	6.97	(1.08, 44.86)	**0.041**	0.41	(0.05, 3.11)	0.390	2.17	(0.48, 9.77)	0.314
Non-menthol	1.00			1.00			1.00		
**Interaction model: menthol x HSI[1]**			0.115			0.360			0.339

*Wave 1 (pre-ban): February-March 2020; Menthol ban: May 2020; Wave 2 (post-ban): September-November 2020; Wave 3 (post-ban): June-July 2021

† Logistic regression models adjusted for flavour of usual brand, sex, age, household income, education, plans to quit within the next six months, ever made a quit attempt, HSI (categorical: 0-3; 4-6), except for the respective stratification covariate;

[1] Interaction models also adjusted for interaction term of flavour of usual brand x respective covariate.

Supplementary Table 6. Comparison of the menthol impact in Canada and in the Netherlands

Table 6a. Comparison of sample characteristics

	**Menthol vs non-menthol**	
**Variable**	**Canada: Chung-Hall et al.**	**Netherlands: Kyriakos et al.**
Region	n/a	ns
Sex	ns	Higher proportion of female menthol smokers, p<.001
Age group	Higher proportion of menthol smokers in 18-24 and 25-39 age groups, p=.016	Higher proportion of menthol smokers in 25-39 age group, p<.001
Ethnicity	ns	n/a
Household income	ns	ns
Education	Higher proportion of menthol smokers in high education group, p=.009	Higher proportion of menthol smokers in high education group, p<.001
Smoking frequency	n/a	Higher proportion of menthol smokers who smoked non-daily, p=.003
Usual brand (FM, RYO)	n/a	Higher proportion of menthol smokers who smoked FM only cigs, p<.001
HSI (mean)	ns	Higher proportion of menthol smokers with lower nicotine dependence, p<.001
Plans to quit in next 6M	ns	Higher proportion of menthol smokers with plans to quit, p=.007
Ever tried to quit	ns	ns
Time-in-sample	ns	n/a

n/a= not applicable; ns=not significant

Table 6b. Comparison of post-ban cessation outcomes by pre-ban menthol smoking status

	**Canada: Chung-Hall et al**	**Netherlands: Kyriakos et al**
**Post-ban quit attempt**		
Pre-ban menthol smokers	58.7%	66.9%
Pre-ban non-menthol smokers	49.0%	49.6%
% diff	9.7	17.3
Chi-square test p-value	p<.05	.002
Adjusted Odd's Ratio	1.61 (1.03-2.51) †	1.89 (1.13-3.16) ††
Logistic regression p-value	p<.05	.015
**Post-ban quit**		
Pre-ban menthol smokers	12.1%	26.1%
Pre-ban non-menthol smokers	5.9%	14.1%
% diff	6.2	12.0
Chi-square test p-value	p<0.05	.002
Adjusted Odd's Ratio	1.30 (0.56-3.01) †	1.63 (0.96-2.88) ††
Logistic regression p-value	ns	ns

†Logistic regression model adjusted for sex, age, household income, education. plans to quit within the next six months, ever made a quit attempt, HSI (categorical: 0-3; 4-6), ethnicity, time-in-sample; †† Logistic regression model adjusted for sex, age, household income, education. plans to quit within the next six months, ever made a quit attempt, HSI (categorical: 0-3; 4-6).

Table 6c. Post-ban transitions among pre-ban menthol smokers

	**Canada: Chung-Hall et al** (n=138 pre-ban menthol smokers)	**Netherlands: Kyriakos et al** (n=99 pre-ban menthol smokers)
Still smoking menthol cigarettes	19.5%	33.0%
Switched to non-menthol cigarettes	59.1%	40.0%
Quit smoking	21.5%	26.1%

BMJ Publishing Group Limited (BMJ) disclaims all liability and responsibility arising from any reliance placed on this supplemental material which has been supplied by the author(s)

Supplementary Table 7. Sensitivity analyses of post-ban quit (Wave 3) by pre-ban menthol status overall using different definitions of quitting and controlling for other covariates, among respondents who participated in Waves 1-3 of the 2020-2021 ITC Netherlands Surveys*, weighted (N=1,322)

	Post-ban quit (Wave 3)						
**Pre-ban menthol use**	**n**	**%**	**95% CI**	**p-value**	**aOR**	**95% CI**	**p-value**
**Definition of quitting in the main text [1]**							
Reported quit or < monthly smoking							
Menthol smoker	26	26.1	(18.3, 35.8)	**0.002**	1.63 †	(0.96, 2.88)	0.090
Non-menthol smoker	175	14.1	(12.3, 16.2)		1.00		
**Other definitions of quitting [2]**							
Reported quit for no minimum length (excludes < monthly smoking)							
Menthol smoker	19	18.9	(12.3, 28.1)	0.052	1.35 †	(0.73, 2.49)	0.334
Non-menthol smoker	150	12.1	(10.4, 14.1)		1.00		
Reported quit for at least one month							
Menthol smoker	16	16.0	(10.0, 24.8)	0.160	1.29 †	(0.68, 2.46)	0.439
Non-menthol smoker	139	11.3	(9.6, 13.2)		1.00		
Reported quit for over 6 months (long-term quit)							
Menthol smoker	11	10.8	(6.0, 18.7)	0.102	1.50 †	(0.71, 3.18)	0.292
Non-menthol smoker	82	6.5	(5.2, 8.0)		1.00		
**Controlling for other covariates [3]**							
Reported quit or less than monthly smoking							
Menthol smoker	26	26.1	(18.3, 35.8)	**0.002**	1.59 ††	(0.76, 3.35)	0.220
Non-menthol smoker	175	14.1	(12.3, 16.2)		1.00		

*Wave 1 (pre-ban): February-March 2020; Menthol ban: May 2020; Wave 2 (post-ban): September-November 2020; Wave 3 (post-ban): June-July 2021

[1] In the main text, smokers were defined as those who were currently smoking cigarettes at least monthly, while quitters were defined as those who self-reported having quit or if they were smoking less than monthly. This allowed comparability between this study and ITC Canada study by Chung-Hall et al., 2021.

[2] Additional sensitivity analyses using different definitions of quitting are presented. Statistical significance attenuated for chi-square tests and regression analyses remained not significant. The overall conclusions reported in this study and the implications remain the same.

[3] In the main text we adjusted for the same covariates used in the ITC Canada study by Chung-Hall et al., 2021 (except for ethnicity, which was not measured in the ITC Netherlands Surveys and time-in-sample which was not applicable since all respondents participated in all three waves) for direct comparability. However, in the presented analysis in this Supplementary table, covariates were included (or not) based on an iterative approach that also considered Akaike (AIC) and Bayesian information criteria (BIC). Added covariates included: daily/non-daily smoking; usual cigarette brand factory-made or roll-your-own tobacco. Covariates considered but not included were: household income, and ever made a quit attempt. Heaviness of Smoking Index (HSI) was controlled for as a continuous variable rather than as a categorical variable (0-3; 4-6).

† Logistic regression model adjusted for sex, age, household income, education. plans to quit within the next six months, ever made a quit attempt, HSI (categorical: 0-3; 4-6).

†† Logistic regression model adjusted for sex, age, education, plans to quit within the next six months, and HSI (continuous, 0-6), daily/non-daily smoking, usual cigarette brand factory-made or roll-your-own tobacco
